# Special Issue on Drug–Membrane Interactions

**DOI:** 10.3390/membranes11100764

**Published:** 2021-10-01

**Authors:** Marina Pinheiro

**Affiliations:** LAQV-REQUIMTE, Departamento de Ciências Químicas, Faculdade de Farmácia, Universidade do Porto, Rua de Jorge Viterbo Ferreira 228, P-4050-313 Porto, Portugal; mpinheiro@ff.up.pt

Drug–membrane interactions immediately occur when drugs are administered, independently of the route of administration or the target location (i.e., intracellular or within the membrane) [1]. The membranes can be extracted from cells or can be imitated using membrane models, which include Langmuir monolayers, liposomes, micelles, and supported lipid bilayers [2,3]. The plasma membrane can generally be mimicked using zwitterionic phospholipids, but also using other lipids and proteins to construct more complex and more realistic membrane models [4]. Other membranes and barriers can also be mimicked using specific components and different charges to mimic organelles (e.g., mitochondria), membranes of specialized cells (e.g., cardiomyocytes), and even membranes of bacteria (Gram-positive, Gram-negative and *Mycobacterium*) [4]. Different biophysical methods can be used to understand drug–membrane interactions and gain better knowledge about the drugs’ pharmacokinetic, pharmacodynamic and toxicity, and consequently, potential side effects of drugs [1]. Methods to assess drug–membrane interactions basically aim to study the partition of the drug to the membrane models, the location of the drug within the lipid bilayers, and the influence of the drug on the biophysical parameters of the membrane models chosen. The techniques used include spectrophotometry, infrared, fluorescence, dynamic light scattering, anisotropy, X-ray, and electron microscopy, among others, and these techniques allowed us to study the interactions at the molecular level, i.e., angstrom (Å) level [1]. Thus, using this small scale we can infer drugs’ affinity to different membranes, their ability to promote changes in the biophysics of the membranes and permeability, and ultimately their potential to lead to membrane disruption and pore formation.

In this Special Issue entitled “*Study on Drug-Membrane Interactions”*, several elegant contributions on drug–membrane interaction studies field were provided. The Special Issue contains seven articles: five research articles and two reviews. The complete description of each study and the main results are presented with more detail in the full manuscript that the reader is invited to explore.

In the study of Ferreira et al. [5], the authors tested a peptide W-BP100 to fight bacterial infections. The developed compound was derived from BP100, holding an additional tryptophan at the N-terminus. Such a small chemical difference was responsible for pronounced differences in the drug–membrane interaction studies. Thus, in contrast to BP100, almost no aggregation of anionic vesicles was observed around saturation conditions. With this study, it was possible to conclude that the incorporation of a single tryptophan to the N-terminus leads to a highly active peptide.

In the study of Pereira-Leite et al. [6], the authors studied two important nonsteroidal anti-inflammatory drugs (NSAIDs): diclofenac as example of one of the most cardiotoxic, and naproxen, which is associated with a low cardiovascular toxicity. The authors concluded that both drugs were able to interact with lipid bilayers and change their permeability and structure. In addition, the authors argue with this study and present the hypothesis that NSAID–lipid interactions, at the mitochondrial level, may be an important step among the mechanisms underlying NSAID-induced cardiotoxicity.

Aguiar et al. [7] studied TP10 peptide conjugates with intrinsic antimalarial activity and their interactions with membrane model membranes of both *Plasmodium*-infected and non-infected erythrocytes. The results of this study confirm that a strong membrane-disruptive character underlies the hemolytic properties of the conjugates, thus hampering their ability to exert selective antimalarial action occurred.

Ferreira et al. [8], studied the interactions of five fluoroquinolones (FQs) and their metalloantibiotics with membranes and demonstrated the role of OmpF porin in the influx. The drug–porin interaction revealed similar values for the association constants of the antibiotics and metalloantibiotics with native OmpF. The authors concluded that free antibiotics exhibited a specific association, with preference for residues on the center of OmpF, whereas the metalloantibiotic exhibited a random interaction. With this study, the authors suggest the use of metalloantibiotics as alternatives to fluoroquinolones to overcome some antimicrobial resistance mechanism of Gram-negative bacteria.

Sohrabi et al. [9] conducted a molecular dynamics software study to simulate the interactions of carbon nanotubes and cell membranes. Dynamics equations for carbon nanotubes were defined in the time and frequency domain using control theory methods. The authors developed a delivery system that consisted of two main parts: crossing through the cell membrane and targeting inside the cell. The designed system provides criteria for crossing through the cell membrane within 30 s to 5 min and a translocation profile of 1 to 100 Å.

The review by Rui et al. [10] is an overview of amino acid derivatives investigated so far as permeation enhancers for the delivery of hydrophilic and lipophilic drugs across the skin, focusing on the structural features which promote their enhancement capacity.

Finally, Cortés et al. [11] reviewed the potential of chitosan-decorated nanoparticles to cross the blood–brain barrier and therefore their promising use in treatment of Alzheimer’s disease, Parkinson’s disease, gliomas, cerebral ischemia, and schizophrenia.

In conclusion, in this Special Issue, we have collected important contributions on different classes of drugs and the correlations between drug–membrane interactions and toxicity and mechanisms of action. Moreover, these contributions also highlighted the high impact on drug–membrane interaction repercussions of a small change in the chemical structure and the utility of “*drug-membrane interaction studies*” for the development of new drugs to fight different classes of diseases, including infectious, inflammatory, and neurological diseases. Finally, in this Special Issue, the concept of the membrane has been presented in a more inclusive perspective, including basement and highly specialized membranes, such as the blood–brain barrier and skin.
